# Targeting *KRASG12D* mutation in non-small cell lung cancer: molecular mechanisms and therapeutic potential

**DOI:** 10.1038/s41417-024-00778-4

**Published:** 2024-05-11

**Authors:** Yining Tang, Xi Pu, Xiao Yuan, Zhonghao Pang, Feng Li, Xu Wang

**Affiliations:** 1 Department of Radiation Oncology, Cancer Institute of Jiangsu University, Affiliated Hospital of Jiangsu University, Zhenjiang, 212000 Jiangsu China; 2 Department of Thoracic Surgery, Affiliated Hospital of Jiangsu University, Zhenjiang, 212000 Jiangsu China

**Keywords:** Targeted therapies, Molecular biology

## Abstract

Lung malignant tumors are a type of cancer with high incidence and mortality rates worldwide. Non-small cell lung cancer (NSCLC) accounts for over 80% of all lung malignant tumors, and most patients are diagnosed at advanced stages, leading to poor prognosis. Over the past decades, various oncogenic driver alterations associated with lung cancer have been identified, each of which can potentially serve as a therapeutic target. Rat sarcoma *(RAS)* genes are the most commonly mutated oncogenes in human cancers, with Kirsten rat sarcoma *(KRAS)* being the most common subtype. The role of *KRAS* oncogene in NSCLC is still not fully understood, and its impact on prognosis remains controversial. Despite the significant advancements in targeted therapy and immune checkpoint inhibitors (ICI) that have transformed the treatment landscape of advanced NSCLC in recent years, targeting KRAS (both directly and indirectly) remains challenging and is still under intensive research. In recent years, significant progress has been made in the development of targeted drugs targeting the NSCLC *KRASG12C* mutant subtype. However, research progress on target drugs for the more common *KRASG12D* subtype has been slow, and currently, no specific drugs have been approved for clinical use, and many questions remain to be answered, such as the mechanisms of resistance in this subtype of NSCLC, how to better utilize combination strategies with multiple treatment modalities, and whether KRASG12D inhibitors offer substantial efficacy in the treatment of advanced NSCLC patients.

## Introduction

Lung cancer is the most common and deadly cancer worldwide [1]. Non-small cell lung cancer (NSCLC) accounts for ~80% of all lung cancers, with lung adenocarcinoma (LUAD) being the most common histological subtype, often accompanied by oncogenic driver mutations [2]. With the development of molecular targeted therapy, some LUAD patients can benefit from specific drugs targeting multiple gene loci, such as *EGFR*, *ALK*, *ROS1*, etc. The RAS proteins (H-RAS, K-RAS, and N-RAS) are GTPases that regulate various cellular activities, including proliferation, survival, growth, migration, differentiation, and cytoskeletal dynamics, through signal transduction pathways. When mutated, RAS proteins remain activated, leading to excessive activation of downstream signaling pathways and ultimately triggering tumor formation. Among them, *KRAS* gene mutations are found in ~30% of lung adenocarcinomas (LUAD). Unlike lung cancers with mutated *EGFR* and *ALK* expressions, *KRAS* has long been considered a challenging therapeutic target, even regarded as “undruggable”. It is generally believed that the difficulty in developing direct KRAS inhibitors is due in part to the pico-molar affinity of GTP and GDP for KRAS (which have much higher cellular concentrations) and to the lack of suitable deep pockets for conformational regulation [3]. Therefore, the development of targeted drugs for KRAS has been difficult for many years. This dilemma was broken in 2013 when American chemical biologist Kevan Shokat discovered that when KRASG12C protein is in the inactive GDP-bound state, the switch II domain (SIIP) of its mutated cysteine residue exhibits a pocket structure that can covalently bind to small molecule drugs [4]. These targeted drugs are now collectively known as KRASG12C inhibitors, including Sotorasib (AMG 510) from Amgen and Adagrasib (MRTX 849) from Mirati, used to treat non-small cell lung cancer patients with *KRASG12C* mutations [5]. Although KRASG12C inhibitors need further research on drug resistance and toxic side effects, their emergence has been a significant breakthrough in the field of KRAS-targeted therapy [6]. *KRASG12D*, as the most common mutation (33%) in *KRAS*-mutant tumors, has its unique molecular mechanism and clinical features, and targeted therapies against it are still under continuous research. Despite significant clinical advancements in targeted treatments, meeting the clinical demands for *KRASG12D* mutation remains challenging to date.

## Characteristics of *KRAS* mutations in NSCLC

### Molecular background

Kirsten-rat-sarcoma viral oncogene homolog (*KRAS*) is the most common mutation driving factor, accounting for 15–30% of all human malignant tumors, and *KRAS* mutations are particularly common in pancreatic ductal adenocarcinoma, colorectal cancer, and NSCLC [7]. Its gene product was initially discovered as p21 GTPase. The conformation of KRAS protein cycles between two different conformational states. When KRAS protein binds to GTP, it is in an active state, and when it binds to GDP, it is in an inactive state. KRAS, in its active state, binds to GTP and has an intrinsic enzymatic activity to cleave the terminal phosphate of nucleotides, converting it to GDP. Its conversion rate is usually slow but can be significantly increased with the assistance of Guanosine triphosphatase-activating protein (GAP). Meanwhile, KRAS can bind to guanine nucleotide exchange factors (GEFs) (such as SOS), causing the bound nucleotide (GDP) to be released and binding of KRAS to GTP [8]. In normal mammalian cells, endogenous KRAS protein mainly exists in an inactive state. However, the oncogenic mutation of KRAS protein interferes with GTP hydrolysis, causing the protein to remain in an active GTP state and continuously transmit signals to the downstream pathway to recruit and activate the proteins required for growth factor and other receptors (such as RAF and PI3K) signal transduction [8].

Furthermore, with the deepening research in recent years, the focus of gene studies has shifted toward non-coding RNAs that play regulatory roles. Among them, long non-coding RNAs (lncRNAs) are a class of non-coding transcripts with a length exceeding 200 nucleotides (nt), and involved in many physiological and pathological processes. Yang et al. reported on *HIF1A-As2*, a *KRAS*-responsive long non-coding RNA (lncRNA), confirming its positive correlation with KRAS through RT-qPCR. Further experiments revealed that *HIF1A-As2* guides a key member of the DExD/H-box helicase family, DHX9, to the promoter region of the oncogenic transcription factor gene *MYC*, thereby enhancing MYC signaling transduction. Activated MYC further promotes cell proliferation and migration in *KRAS*-driven NSCLC. Simultaneously, KRAS, through MYC, promotes *HIF1A-As2*, forming a positive feedback loop [9]. MicroRNAs (miRNAs) are another important class of non-coding RNAs that play a role in gene regulation by degrading mRNA or inhibiting translation. Shi et al. used NanoString technology and Real-time PCR to identify the most upregulated microRNAs (miR-30c and miR-21) in cells overexpressing KRASWT and KRASG12D. Through experiments, they demonstrated that miR-30c downregulates BID, NF1, RASA1, and RASSF8 at the transcriptional level, while miR-21 inhibits the protein expression of RASA1 and RASSF8 to contributes to tumorigenesis [10].

### Incidence

Statistics on *RAS* mutations in 17,993 cancer patients showed that the total mutation frequency of *RAS* genes was 22.58%, with *KRAS* mutations being the most frequent and most commonly observed in pancreatic cancer (685/842, 81.35%), colorectal cancer (85/175, 48.57%), and colon cancer (1609/3329, 48.33%) [11]. *KRAS* mutations are concentrated in codons 12 and 13 of exon 2 and codon 61 of exon 3. The most common mutation subtypes in *KRAS*-mutant lung adenocarcinoma are codon 12 substitution mutations (purine is replaced by pyrimidine or vice versa) *G12C* (39%) and *G12V* (18–21%), followed by transition mutations (purine is replaced by purine or pyrimidine is replaced by pyrimidine) *G12D* (14–18%) and *G12A* (10–11%). *G12C* is the most common mutation (45%) in former/current smokers, and *G12D* is the most common mutation (46%) in never-smokers [12]. In addition, *KRAS* also shows a different frequency depending on the patient’s race (more common in white populations than Asians). A multicenter retrospective cohort study of 216 Asian *KRAS*-mutant NSCLC patients analyzed by Lee et al. found that most patients were male (70.8%), Eastern Cooperative Oncology Group (ECOG) physical status scores were mostly 0–1 (92.1%), histological subtypes included adenocarcinoma (89.8%), squamous cell carcinoma (4.2%), and others (6.0%), and *KRASG12D* was the most common subtype (25.5%), mostly in never-smokers, suggesting that *KRAS*-mutant lung cancer in Asian patients may be driven by factors other than smoking [13]. Cooper et al. studied the clinical characteristics of *KRASG12D*-mutant NSCLC and found that among 107 *KRASG12D*-mutant NSCLC patients, most had a history of smoking (80 cases, 74.8%), and the histological subtype of the tumor was mostly adenocarcinoma (93 cases, 86.9%). Coexisting mutations were more common in *KRASG12D*-mutant NSCLC, including *STK11* (17/107, 15.9%), *TP53* (36/107, 33.6%), and *KEAP1* (10/107, 9.4%). The co-occurrence of *STK11* and *KEAP1* mutations was associated with a poor clinical outcome, while the co-occurrence of *TP53* did not affect survival [14]. Chen et al. analyzed 18,224 *KRAS*-mutant NSCLC patients to investigate the clinical characteristics of *KRAS*-mutant NSCLC in China. Among them, *G12C* (29.6%) was the most common subtype, followed by *G12D* (18.1%) and *G12V* (17.5%). The highest incidence of co-mutation was with *TP53* (33.6%), followed by *EGFR* (11.6%), *STK11* (10.4%), *KEAP1* (6.2%), and *CDKN2A* (6.0%) [15].

### The prognostic

The impact of *KRAS* mutations remains controversial, with inconsistent findings in various studies [16]. Wahl et al. investigated the correlation between *KRAS* status (*KRAS* wt vs. *KRAS* mut), *KRASG12* status (*KRAS* wt vs. *KRASG12C* vs. *KRAS* non-*G12C* mutations), and *KRAS* mutation type (*G12C*, *G12V*, *G12D*, and *G12A*) and survival in multivariate analysis, entire cohorts, curative resection patients, or advanced patients, and none of the control groups showed any correlation with survival [12] (Table 1). However, Cai et al. revealed that *KRASG12D* mutation was associated with the shortest progression-free survival (PFS) and overall survival (OS) when compared with *KRASG12C* and *KRASG12V*, and the *KRAS* G > T group had better PFS and OS than the *KRAS* G > C and *KRAS* G > A groups at the amino acid substitution level [1]. Similarly, Johnson et al. found that *KRAS* mutation was an independent factor associated with shorter survival because of inherent biological differences in *KRAS*-driven lung cancer rather than differences in receiving prolonging life treatments like platinum-based chemotherapy and bevacizumab [17]. Aredo et al. explored the impact of *KRAS* mutation and co-mutations in the prognosis of NSCLC patients and found that *KRASG12D* mutation was significantly associated with OS in multivariate analysis, and *STK11* co-mutation was also significantly associated with OS [18]. Arbour et al. identified different biological subtypes of *KRAS*-mutant lung adenocarcinoma that could be associated with co-mutations and investigated the effect of co-mutations on patient prognosis and treatment response. For the 330 patients with advanced *KRAS*-mutant lung cancer screened, the most common co-occurring genomic alterations were *TP53* (42%), *STK11* (29%), and *KEAP1/NFE2L2* (27%), and *KEAP1/NFE2L2* co-mutation was an independent prognostic factor that predicted shorter survival [19]. Shepherd et al. summarized the prognostic and predictive roles of *KRAS* mutation status and subtype in early-stage resected NSCLC in four adjuvant chemotherapy trials and found that *KRAS* mutation status was not a significant independent prognostic factor, especially for amino acid substitution mutations in the 12th codon [20]. Yu et al. retrospected 677 patients with metastatic or recurrent *KRAS*-mutant lung cancer and evaluated the relationship between *KRAS* mutation type, clinical factors, and overall survival. The data showed that the median overall survival for all patients with *KRAS*-mutant advanced lung cancer was 1.2 years, and the median overall survival ranged from 0.7 years (*G13C*) to 1.5 years (*G12F*) for specific *KRAS* point mutations, and no significant differences in survival were observed when comparing different *KRAS* point mutations [21]. Chen et al. treated 497 *KRAS*-mutant patients with different combinations of chemotherapy drugs and with or without immune checkpoint inhibitors (ICIs) and found no significant differences in PFS among the most common three subtypes of *G12C*, *G12D*, and *G12V*, with median PFS of 5.7, 6.6, and 6.6 months, respectively. The data also indicated that the patients who received ICI plus chemotherapy had significantly longer survival than those who received monotherapy [15].

**Table 1:** *KRAS* status as a predictive biomarker for lung cancer.

Reference	Patients tested for *KRAS*	Patients by *KRAS* status		Treatment arm	Endpoint	*KRAS*-mut	*KRAS*-wt	Conclusion
		*KRAS*-mut	*KRAS*-wt					
Shepherd et al. [20]	1543 (not specified)	300	1243	Cisplatin-based ACT	OS	NA	NA	No difference. Similar OS among *KRAS*-mut and *KRAS*-wt
Arbour et al. [19]	330 (advanced stage)	*KRAS* 330	NA	Platinum/pemetrexed + bevacizumab	OS(month)	17	NA	There was a significantly shorter survival in patients with co-mutations in *KEAP1/NFE2L2. STK11* and *TP53* co-mutation statuses were not associated with survival.
		*KRAS*/*TP53* 137				17		
		*KRAS*/*STK11* 95				12		
		*KRAS*/*KEAP1 or NFE2L2* 85				10		
Yu et al. [21]	677 (stage IV)	*KRAS(G12)* 624	NA	Platinum-based chemotherapy, pemetrexed and/or bevacizumab	OS(month)	15	NA	No difference. Similar OS among *KRAS* mutant subtypes
		*KRAS(G13)* 53				13		
Wahl et al. [12]	1117 (stage I–IV)	420	697	NA	PFS(month)	16.3	17.1	No difference. Similar OS and PFS among *KRAS* and *KRAS*-wt
					OS(month)	46.5	37.1	
Cai et al. [1]	1126 (advanced stage)	*KRAS(G12C)* 24	NA	Pemetrexed + platinum	PFS(month)	15.57	NA	The PFS and OS of patients with *KRASG12D* mutation were inferior to those with *KRASG12C* mutation or *KRASG12V* mutation.
		*KRAS(G12D)* 20				11.00		
		*KRAS(G12V)* 16				23.28		
					OS(month)	18.64		
Johnson et al. [17]	1036 (advanced stage)	241	520	EGFR-TKIs, platinum, bevacizumab	OS(month)	16	23	*KRAS* mutations were associated with shorter survival
Aredoa et al. [18]	186 (not specified)	*KRAS* 186	NA	NA	OS(month)	36	NA	*KRAS* and *STK11* mutations confer poor prognoses for patients with *KRAS*-mutant NSCLC
		*KRAS/STK11* 22				22		
Chen et al. [15]	497 (stage I–IV)	*KRAS(G12C)* 90	NA	Pemetrexed + bevacizumab	PFS(month)	9.6	NA	Regarding mutated sites of *KRAS*, there was no significant difference in PFS among the most common three mutational sites.
		*KRAS(G12D)* 45				3.8		
		*KRAS(G12V)* 48				10.4		
Passiglia et al. [25]	530 (stage III–IV)	206	324	Nivolumab	PFS(month)	4	3	*KRAS* status is not a reliable predictor of nivolumab efficacy.

*NA* not available, *OS* overall survival, *PFS* progression-free survival, *mut* mutation, *wt* wild type.

### Sensitivity to treatment

Currently, immunotherapy alone or in combination with platinum-based chemotherapy is the standard first-line treatment for *KRAS*-mutant NSCLC, but its efficacy is influenced by multiple factors. Fancelli et al. conducted a retrospective analysis showing that single-agent chemotherapy had limited efficacy in the *KRAS*-mutant NSCLC population, while single-agent ICI or ICI plus chemotherapy could benefit this population, which was not related to PD-L1 overexpression [22]. Sun et al. demonstrated that the clinical outcomes of *KRAS*-mutant NSCLC patients varied in response to ICI-based first-line treatment to some extent with different *KRAS* mutation subtypes and co-mutations. However, patients with *KRAS*/*SMAD 4* co-mutation had a poor prognosis and were considered a new genotype that was insensitive to treatment [23]. Ghimessy et al. analyzed the effect of the *KRAS* mutation subtype on bevacizumab (vascular endothelial growth factor inhibitor) and found that BEV had lower activity in *KRAS*-mutant tumors, especially in *KRASG12D*-mutant stage III–IV LUAD patients than in non-*KRAS*-mutant LUAD patients [24]. Passiglia et al. investigated the efficacy and safety of nivolumab (immune checkpoint inhibitor) in 530 pretreated patients with advanced NSCLC and *KRAS* mutations. The data showed that *KRAS* status did not affect the efficacy of nivolumab in NSCLC, and *TP53* co-mutated NSCLC patients also showed significant and durable clinical benefits from anti-PD-1 therapy, while patients with *LKB1/STK11* tumor suppressor gene co-mutations were associated with shorter PFS and OS, possibly suggesting that *LKB1* deficiency is a major driver of immune escape and a genomic biomarker of innate resistance to ICIs [25].

## Activation of *KRASG12D* mutation-related signaling pathways

**Fig. 1:**
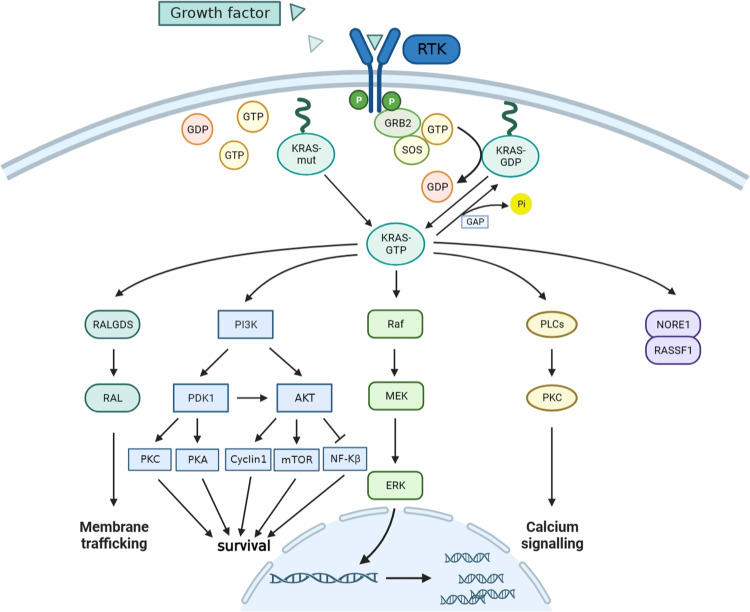
KRAS signaling pathway. Upon activation by receptor tyrosine kinases, the receptors bind with the adapter protein RAS. RAS also activates phosphatidylinositol 3-kinases (PI3Ks) through protein growth factor receptor-binding protein 2 (GRB2). Then, GRB2 binds to participate in the activation of multiple downstream effectors, including SOS1 or 2 (guanine nucleotide exchange factors [GEFs] that cause phosphoinositide-dependent kinase-1 (PDK1) and protein kinase B (PKB, also known as AKT) to be activated by nucleotide exchange and replacement of GDP with GTP. AKT plays an important role in inhibiting NF-KB and multiple RAS functions. Once activated, RAS interacts with multiple effector transcription factors and activates the mammalian target of rapamycin (mTOR). PI3K also activates the monomeric GTPase RAC involved in mitogen-activated protein kinase/extracellular signal-regulated kinase (MEK1 and 2) and catalyzes cell migration. Finally, RAS also activates the RALGDS protein, which is a guanine nucleotide exchange factor for MAPK and RAL and phospholipase C activation, leading to the transcription of multiple transcription factors, including ELK1 and activation protein 1 (AP1). It also activates protein kinase C (PKC) and mobilizes intracellular calcium. RAF protein is essential for the proliferative action of PKA signaling protein kinase A. The mutant KRAS maintains a low level of GTPase activity, resulting in a weak response to upstream signals and a constitutively active state. G12D continuously activates downstream pathways, mainly through the RAF/MEK/ERK signaling pathway, leading to abnormal cell proliferation.

Currently, the KRAS-related signaling pathways have been extensively studied, as shown in Fig. 1. However, the abnormal activation of other proteins in the pathway carried by *KRAS*-mutated NSCLC will affect the treatment and prognosis of these patients. The ERBB family of receptor tyrosine kinases (RTKs), which includes 4 subtypes, can form dimers and bind to a range of ligands, to jointly activate a signaling network driven by ERBB. While KRAS mutations have traditionally been considered independent of upstream regulation, this perspective has been challenged by the work of Kruspig et al. Their research shows that multiple ERBB receptors are expressed and activated from the earliest stages of *KRAS*-driven lung tumor development. Additionally, lung tumors driven by *KRASG12D* mutation express multiple ERBB ligands, and active ERBB enhances signal transduction through the core RAS → ERK pathway, promoting cancer cell proliferation and tumor progression [26]. The RAS-specific guanine nucleotide exchange factors Son of sevenless (SOS) activates RAS by catalyzing the exchange of GDP for GTP, facilitating the activation of RAS. Mutant KRAS reduces its own GTPase-activating protein (GAP) activity, allowing SOS1 to serve as a central hub in regulating KRAS. SOS1 catalyzes the formation of KRAS-GTP complex to activate multiple downstream signaling pathways that trigger cancer cell proliferation. Fernando et al. discovered that the loss of SOS1 impairs the development and progression of *KRASG12D*-driven LUAD lung tumors. Their experiments demonstrated that SOS1 deficiency specifically inhibits the rates of lung tumor cell proliferation (Ki67) and ERK activation (pERK), while also significantly affecting the pro-tumor activities of various cell subpopulations within the LUAD microenvironment [27]. Ihle et al. studied the different effects of *KRAS* mutations on drug sensitivity and the impact on different signaling pathways. They found that NSCLC cell lines carrying the *KRASG12D* mutation have activated phosphatidylinositol 3-kinase (PI3K) and mitogen-activated protein kinase/extracellular signal-regulated kinase (MEK) signaling pathways. The PI3K/AKT signaling pathway is constitutively activated by mutant *KRASG12D* and is not inhibited by mTOR. However, the bulky Asp of *KRASG12D* interferes with the formation of KRAS homodimers and the binding of RALGDS, and downstream effector RAL is not activated. Therefore, the substitution of different amino acids induces heterogeneity in the behavior of KRAS protein, resulting in different signal outputs. This has profound implications for identifying and treating *KRAS*-driven tumors, and different combinations of downstream signaling pathway inhibitors may be required when treating *KRAS* mutation lung cancer with different amino acid substitutions [28]. Hung et al. studied the different mechanisms of RhoA/Wnt-induced NSCLC metastasis in different *KRAS* mutation subtypes. Immunoblotting results showed that *KRASG12D* mutation activates RhoA and further eliminates the activation of Wnt/β-catenin protein signaling, reducing the metastatic activity of *KRASG12D*-mutated NSCLC. This study confirms the relevance of the KRAS/RhoA/Wnt/β-catenin signaling pathway in NSCLC metastasis [29].

## Targeted therapy for *KRASG12D* mutation

For decades, mutated *KRAS* has been recognized as an attractive drug target for treating multiple types of cancer, but the development of targeted drugs has not been as successful as anticipated. The difficulties in targeting KRAS are due to several factors: (1) the broad scope of KRAS’s activity, including its essential role in many normal cellular functions, meaning drugs that directly inhibit KRAS may have significant toxicity and strong side effects; (2) KRAS’s primary functional domain involves a pocket that binds to GDP or GTP. Unlike protein kinases, which have a weak affinity for ATP, KRAS’s binding to GTP or GDP is extremely strong, with an affinity coefficient on the picomolar (10–12) level, while the concentration of GDP and GTP in normal cells is on the micromolar (10–6) level. This means that finding a small molecule compound with a binding ability to KRAS that is equivalent to GDP or GTP is extremely challenging; (3) designing a drug that selectively inhibits the activity of mutated KRAS protein while minimizing the impact on normal KRAS activity requires a compound with good selectivity for mutated KRAS, which is another difficult challenge in drug design; and (4) indirect strategies for targeting KRAS are also fraught with challenges, including the fact that KRAS signaling pathway is a necessary pathway for normal cell growth and survival, and targeting essential pathways is often associated with significant toxicity that reduces the therapeutic window to the point where it may be absent, compensatory escape mechanisms, and feedback and redundancy resulting from strict regulation.

### Direct targeting drugs

#### KRASG12D inhibitors

**Fig. 2:**
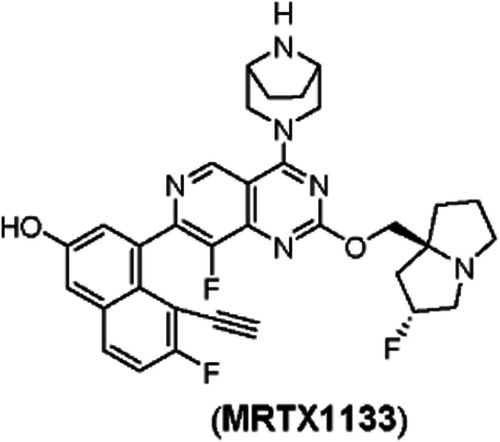
MRTX1133. The chemical structure of the KRASG12D inhibitor MRTX1133.

Wang et al. developed MRTX1133 (Fig. 2), a potent, selective, non-covalent KRASG12D inhibitor with picomolar binding affinity, through a series of structurally-based optimizations on the KRASG12C inhibitor adagrasib. Firstly, the compound has a pyrido[4,3-d]pyrimidine scaffold, and three substituents were searched to interact widely with KRASG12D protein. The C4 position is a [3.2.1]bicyclic diamino substituent to achieve optimal interaction with Asp12 and Gly60 in the mutant. The C2 position is modified with the pyrrolizidine with a 2-fluoro substituent which forms a strong ionic interaction with the negatively charged Glu62 carboxylate salt. Finally, the C7 substituent is an optimized 7-fluoro and 8-ethynyl group that lies well within the hydrophobic pocket of KRASG12D protein and forms a well-organized hydrogen-bonding network. The interaction of hydrogen bonds allows the terminal ethynyl group to effectively bridge the lipophilic and polar regions of the KRASG12D protein switch II pocket. Experimental validation demonstrated that MRTX1133 inhibits KRASG12D signal transduction in cells and in vivo, and its anti-tumor effects have been confirmed in a mouse model, showing robust in vivo efficacy and potential for targeted therapy against this “undruggable” target [30].

Mao et al. used a strategy based on strong interactions (salt bridges) between the alkylamine moiety and Asp 12 on the inhibitor to design a series of potent inhibitors (TH-Z816, TH-Z827, and TH-Z835) that can form salt bridges with the Asp 12 residue of KRASG12D, using the G12C inhibitor MRTX 22 as a scaffold and characterized their in vitro and in vivo activity. ITC experiments showed that these salt bridge-forming inhibitors bound to both GDP and GTP-bound KRASG12D and effectively disrupted KRAS-CRAF interaction, but did not bind to wild-type or G12C mutant KRAS. These molecules also disrupted the activation of MAPK and PI3K/mTOR signaling in different cancer cells and displayed anti-proliferative and anti-tumor effects. This study demonstrated proof-of-concept for the strategy of targeting KRASG12D by inducing adaptation pockets via salt bridge formation [3].

Meanwhile, Zhou et al. discovered an effective, selective, biologically stable, and cell-permeable peptide drug, NKTP-3, which targets NRP1 and KRASG12D. NKTP-3 first binds to NRP1 on the cancer cell membrane and is then delivered into the cell. Once inside the cell, it binds to KRASG12D and significantly inhibits downstream signaling, including AKT and ERK phosphorylation, leading to anti-tumor effects. Strong anti-tumor activity of the NRP1/KRASG12D dual-targeting cyclic peptide NKTP-3 was demonstrated in xenografts derived from A427 cells and primary lung cancer model driven by KRASG12D, with no obvious toxicity. These findings suggest that NKTP-3 may be a potential drug for treating *KRASG12D*-driven lung cancer [31].

In 2017, a synthetic cyclic peptide, KRpep-2d, was discovered as the first selective inhibitor of KRASG12D. The two Cys residues in the peptide are essential for its cyclic structure and control of its binding and inhibitory activity, but the bond is cleaved under intracellular reducing conditions, limiting application. Sakamoto et al. generated KS-58, a KRpep-2d derivative identified as a bicyclic peptide with a non-proteinogenic amino acid structure. KS-58 enters cells and exerts anti-cancer effects by blocking two pathways: RASGDP-SOS1 interaction (i.e., GDP-GTP exchange on RAS) and RASGTP-BRAF interaction. KS-58 was shown to selectively bind to KRASG12D and inhibit the in vitro proliferation of both the A427 human lung cancer cell line and the PANC-1 human pancreatic cancer cell line that expresses *KRASG12D*. However, the pharmacokinetic properties and high doses required for the treatment of this drug still need improvement. Nevertheless, KS-58 is an attractive lead molecule for developing new cancer drugs that target KRASG12D [32].

#### Pan-KRAS inhibitors

The broad-spectrum KRAS inhibitor is defined as a non-covalent inhibitor that exhibits high affinity for the inactive state of KRAS and can block nucleotide exchange to prevent the activation of wild-type KRAS and a wide range of KRAS mutants [33]. Using the selective KRASG12C inhibitor BI-0474 as a starting point, Kim et al. designed a broad-spectrum KRAS inhibitor, BI-2865, with potent non-covalent inhibitory activity. Experimental evidence showed that this inhibitor demonstrated similar efficacy to BI-0474 against KRASG12C mutant cells while also significantly inhibiting cell proliferation in G12D or G12V mutant cells. The inhibitor functions by preferentially targeting the inactive state of KRAS to prevent its reactivation through nucleotide exchange. Additionally, the research group conducted experiments with BI-2865 to inhibit NRAS and HRAS mutant cells, revealing that the inhibitor’s ability to inhibit nucleotide exchange in HRAS or NRAS is several orders of magnitude lower than that in KRAS, and this difference is attributed to direct and/or indirect constraints imposed by three residues in the G domain. Consequently, in cells with wild-type *KRAS*, the use of this inhibitor leads to increased activation of other RAS homologs, thereby limiting its antiproliferative effects [33].

SOS1 is a key guanine nucleotide exchange factor (GEF) for KRAS, which binds to KRAS protein at its catalytic binding site and promotes the exchange of GDP for GTP, thereby activating the KRAS protein. In addition to its catalytic site, SOS1 can also bind to GTP-bound KRAS at an allosteric site, forming a positive feedback regulation mechanism [34]. Hofmann et al. reported the discovery of a highly efficient, selective, and orally bioavailable small molecule SOS1 inhibitor BI-3406, which binds to the catalytic domain of SOS1, thereby preventing its interaction with KRAS. Experimental evidence suggests that BI-3406 reduces the formation of GTP-loaded KRAS and restricts the growth of most tumor cells driven by *KRAS* variants at positions G12 and G13. Furthermore, BI-3406 can weaken the feedback reactivation induced by MEK inhibitors, thereby enhancing the sensitivity of *KRAS*-dependent cancers to MEK inhibition. Thus, the development of clinical SOS1 compounds in combination with MEK inhibitors and potentially other RTK/MAPK pathway inhibitors holds promise for significant clinical benefits [35]. Hillig et al. designed a pan-KRAS inhibitor, BAY-293, using a dual-screening approach and structure-guided design. The study demonstrated that this inhibitor binds to a surface pocket on SOS1, preventing the formation of the KRAS-SOS1 complex. This pocket is located adjacent to the KRAS binding site and thus blocks the reloading of KRAS with GTP, leading to anti-proliferative activity. BAY-293 also exhibits synergistic effects with covalent inhibitors of KRASG12D, highlighting the potential of combined therapy targeting both KRAS and SOS1 [36].

### Indirectly targeted drugs

#### MEK inhibitor

Due to the difficulty of directly targeting KRASG12D drugs, targeting the KRAS signaling pathway has always focused on downstream targets, one of which is MEK. Drugs targeting MEK downstream in the MAPK cascade via inhibition of signal transduction pathways are less effective in treating *KRAS*-mutant NSCLC in multiple experiments. For example, Pasi et al. found that the addition of the MEK inhibitor trametinib to docetaxel did not improve progression-free survival in advanced *KRAS*-mutant non-small cell lung cancer patients compared to docetaxel alone [37]. The main reason is that although targeting MEK blocks MAPK cascade signaling, other downstream pathways of KRAS (such as PI3K-AKT, RAL, etc.) are strengthened. Lee et al. demonstrated synergistic effects of combination therapy with MEK inhibitor cobimetinib and immunotherapy for the treatment of *KRAS*-mutant NSCLC, showing anti-tumor effects and improved survival in a mouse model [38].

#### GRP78 inhibitor

Studies have shown that newly synthesized KRAS is cytoplasmic and inactive and undergoes a series of translation and post-translational modifications on the cytoplasmic surface of the endoplasmic reticulum (ER), which are mediated by enzymes that act as transmembrane ER proteins. Therefore, ER is the main site of KRAS maturation, and perturbation of ER homeostasis and protein quality control may be detrimental to *KRAS*-driven LUAD. The 78-kDa glucose-regulated protein GRP 78/BiP is a critical chaperone protein in the ER and a major pro-survival effector in the unfolded protein response (UPR). The loss of GRP78 induces UPR and apoptotic markers, which are associated with the loss of cell viability in lung cancer cell lines carrying the same *KRAS* mutation [39]. Ha et al. targeted GRP78 with small molecule inhibitors (such as HA15 and YUW70) with anti-cancer activity, which consistently reduced the levels of oncogenic KRAS protein in the tested cell lines. They also found that GRP78 deficiency can inhibit PI3K, AKT, TGF-β, and CD44 signaling pathways, as well as many other signaling pathways. Combined with ER stress-induced cell apoptosis and autophagy, this will provide a strong defense against the development of cancer cell resistance before cancer cells are eliminated [40].

#### NFkB activating kinase inhibitor

Preclinical studies have provided evidence that both classical and non-classical NF-κB pathways are co-activated in LUAD-carrying *KRAS* mutation. The specificity of IKK (NFkB activation kinase) synergistically induces tumorigenesis with mutant KRAS in an autocrine manner, providing survival advantages for mutant cells in vitro and in vivo. The NCT01833143 phase II single-center clinical trial of bortezomib subcutaneously administered to patients with advanced NSCLC carrying *KRASG12D* mutations or without a previous smoking history at Memorial Sloan Kettering Cancer Center showed some anti-tumor activity, especially in a unique subtype of lung adenocarcinoma, invasive mucinous adenocarcinoma (IMA), while boron-tazoxime is inactive in most patients with advanced *KRASG12D* mutant lung adenocarcinomas. Therefore, novel inhibitors of the NF-κB pathway need to be explored [41].

#### HSP inhibitor

Inhibiting heat shock proteins has been identified as another potential therapeutic strategy for *KRAS*-mutant NSCLC. Molecular chaperone Hsp 90 is essential for protein stability and maturation and prevents protein degradation by the proteasome. Vreka et al. found that IKKα is a partner of *KRAS* non-oncogene addiction, and specifically synergizes with mutant KRAS to induce tumorigenesis, providing survival advantages for mutant cells in vitro and in vivo. The Hsp 90 inhibitor 17-DMAG can block IKK function and have better efficacy against *KRASG12D*-mutant lung adenocarcinoma, opening up a new way to prevent/treat *KRAS*-mutant LUAD [42].

#### ERBB inhibitors

Previous research has shown that *EGFR* mutations and *KRAS* mutations rarely occur together, and the use of EGFR-targeted drugs alone to treat *KRAS*-mutant lung adenocarcinoma has not shown significant clinical benefits. However, recent experimental results suggest that the independence of mutated KRAS from upstream signaling pathways may not be absolute. Kruspig et al. demonstrated through experiments that the initiation and progression of *KRAS*-driven lung tumors require the involvement of ERBB family receptor tyrosine kinases (RTKs), and inhibition of the ERBB network weakens the activation of a series of downstream signaling proteins (such as pERK, STAT3, etc.), while transient pharmacological inhibition of the ERBB network enhances the therapeutic benefits of MEK inhibitors in the autologous tumor environment. Multiple ERBB inhibitors almost completely inhibit the formation of *KRASG12D*-driven lung tumors and enhance the benefits of MEK inhibition in tumor therapy [26].

#### SHP2 inhibitors

SHP2, encoded by the *PTPN11* gene, plays an important role in signal transduction downstream of growth factor receptors by mainly regulating cell survival and proliferation through activation of the RAS-ERK signaling pathway. Ruess et al. found that *PTPN11* gene deletion significantly inhibited tumor development in *KRAS*-driven pancreatic ductal adenocarcinoma and non-small cell lung cancer mouse models, providing evidence for the critical dependence of mutant *KRAS* on SHP2 in the process of carcinogenesis [43]. Chen et al. found that SHP2 is activated by peptides and proteins containing appropriately spaced phosphotyrosine residues, which bind the N-terminal and C-terminal SH2 domains in a bidentate manner, releasing it from the self-inhibitory interface, and make the active site available for substrate recognition and turnover. They used this natural regulatory mechanism to screen out the SHP2 inhibitor SHP099, which locks SHP2 in an autoinhibitory conformation and directly targets the inhibition of MAPK signaling and proliferation in RTK-dependent cells. This provides a feasible strategy for targeting RTK for cancer treatment [44]. Nichols et al. found through the treatment with another SHP2 inhibitor, RMC-4550 (a small molecule allosteric inhibitor), that it reduces oncogenic RAS-RAF-MEK-ERK signaling and cancer growth by disrupting SOS1-mediated RAS-GTP loading, highlighting SHP2 inhibition as a promising molecular therapeutic strategy for nucleotide cycling oncogenic KRAS in cancer [45].

#### Immune therapies

The inhibition and rewiring of the immune system play a crucial role in the onset and development of tumors. Immune therapies aim to reactivate anti-tumor immune cells and overcome the tumor’s immune escape mechanisms. Tumor immunotherapy, represented by immune checkpoint blockade and adoptive cell transfer, has made enormous clinical successes by inducing long-term remission of some tumors that are difficult to treat with all other therapies. Among them, immune checkpoint blockade therapy represented by PD-1/PD-L1 inhibitors (nivolumab) and CTLA-4 inhibitors (ipilimumab) has shown encouraging therapeutic effects in the treatment of various malignancies, such as non-small cell lung cancer (NSCLC) and melanoma, etc. [46].

Immune checkpoint inhibitors (ICIs), mainly represented by PD-1/PD-L1 inhibitors (nivolumab), have been widely used in the treatment of non-small cell lung cancer (NSCLC). ICIs are currently used as single-agent therapy or in combination with other treatments for first-line and subsequent therapy of metastatic NSCLC. In addition, ICI in the neoadjuvant and adjuvant treatment setting has shown efficacy for resectable disease patients, highlighting the potential of ICIs to improve outcomes for this patient group [47]. However, research has shown that most tumors have immune inhibitory mechanisms that limit the effectiveness of immunotherapy, including PD-1 expression on tumor-infiltrating T cells and the accumulation of inhibitory T cells, such as CD4^+^Foxp3^+^ regulatory T (Treg) cells, in the tumor microenvironment, which hinders anti-tumor immune responses. Studies have shown that *KRASG12D* mutation correlates with reduced TMB, and *KRASG12D/TP53* co-mutation has a significant effect on reducing TMB and PD-L1 expression and reducing immune cell infiltration. *KRASG12D* mutation, especially in combination with *TP53* co-mutation, maybe a negative predictive biomarker for PD-1/PD-L1 immune checkpoint inhibitors in NSCLC patients [48]. Eliminating these inhibitory mechanisms in tumors can pave the way for more effective anti-tumor responses.

Martinez-Usatorre et al. improved the efficacy of PD-1/PD-L1 inhibitors by modulating the tumor microenvironment in a *KRASG12D*^/+^;*TP53*^−/−^ genetically engineered mouse model. They induced vascular normalization and facilitated T cell trafficking through inhibition of angiogenic factors VEGFA and ANGPT2 with A2 V, which improved maturation and antigen presentation of tumor-associated macrophages (TAMs). They also used CSF1R inhibitor 2G2 and platinum-based chemotherapy to deplete TAMs and reduce Treg cell numbers, respectively, to enhance the response of *KRAS* tumors to A2 V and anti-PD-1 dual therapy. The combination of these three agents induced an immune-infiltrated tumor microenvironment with increased CD4^+^ and CD8^+^ T cells, and decreased TAMs and Treg cells, leading to improvement in the response of KP tumors to checkpoint inhibitors [49]. Adeegbe et al. improved the response of *KRAS*-mutant NSCLC to immune therapy by combining JQl (a BET family inhibitor containing a bromodomain) with anti-PD-1 treatment. Bromodomain proteins are epigenetic regulators that cause growth inhibition and/or cell cytotoxicity in tumor cells. Treatment with JQl alone induced a decrease in Treg cell numbers, while combination with anti-PD-1 enhanced activation of infiltrated T cells in the tumor bed and improved effector function, leading to increased expression of Th1 cytokine profile, which is consistent with the persistent antitumor response observed with this novel treatment combination [48]. In addition, Lee et al. improved the prognosis of *KRAS*-driven lung cancer by combining MEK inhibitors with immunomodulatory anti-PD-1 and anti-PD-L1 antibodies. Low-dose MEKi (trametinib) and anti-PD-L1 combined therapy in *KRAS*-mutant NSCLC enhanced tumor microenvironment and T cell infiltration, reduced Ly6G^high^ PMN-MDSCs (myeloid-derived suppressor cells, a type of immune inhibitory cells that mainly suppress natural killer cells and effector CD8^+^ T cells) in tumor tissue, and inhibited tumor cell proliferation, leading to tumor cell apoptosis. MEKi acted as a sensitizer for *KRAS*-mutant tumors that were previously unresponsive to immune therapy [38].

## Conclusion

In the context of advanced NSCLC, targeted therapy against driver oncogenic mutations has already changed the treatment paradigm. Given the high incidence of *KRAS* mutations in NSCLC patients, this is a promising treatment target. The 2023 NCCN guidelines for the first time recommended KRASG12C targeted drug Adagrasib for the treatment of *KRASG12C* mutant NSCLC, which is a significant breakthrough for KRAS treatment targets. However, other coexisting mutations and changes in the immune microenvironment may be critical for its function and biological impact. Therefore, combination therapy with chemotherapy, immunotherapy, targeted therapy, and other treatment modalities may further improve the poor prognosis of *KRAS*-mutant NSCLC. Moreover, as *KRAS* mutation involves multiple downstream signaling pathways, targeting multiple different targets with combination targeted therapy may improve the current inadequate therapeutic efficacy of targeted drugs. Shortly, new molecules or treatment strategies may radically alter the outcome of patients with *KRAS*-driven NSCLC. Further research is required to better understand the pathways involved in *KRAS* mutation and to develop more targeted and effective drugs.
